# Editorial: Exploring molecular recognition: integrating experimental and computational approaches

**DOI:** 10.3389/fmolb.2025.1637793

**Published:** 2025-06-10

**Authors:** Teresa Matamá, António Rego, Junmei Wang, Tarsila G. Castro

**Affiliations:** ^1^ Center of Biological Engineering, University of Minho, Campus de Gualtar, Braga, Portugal; ^2^ Department of Biology, School of Sciences, University of Minho, Braga, Portugal; ^3^ Department of Pharmaceutical Sciences, University of Pittsburgh, Pittsburgh, PA, United States; ^4^ SOLFARCOS, Soluções Farmacêuticas e Cosméticas, Lda., Braga, Portugal

**Keywords:** molecular recognition, molecular interaction, signal transduction, therapeutic drug target identification, AI/machine learning

Molecular recognition, the process by which molecules interact through noncovalent forces, underpins the foundation of cellular function and human health. In biological systems, biomolecules such as proteins, nucleic acids, carbohydrates, and lipids bind to one another with remarkable specificity. These interactions drive essential processes, including signal transduction, metabolic regulation, gene expression, and immune responses. The strength and accuracy of these interactions ensure proper cellular communication and homeostasis. Disruptions in molecular recognition often lead to disease, which highlights its importance in health and therapeutic development (Figure 1).

**FIGURE 1:**
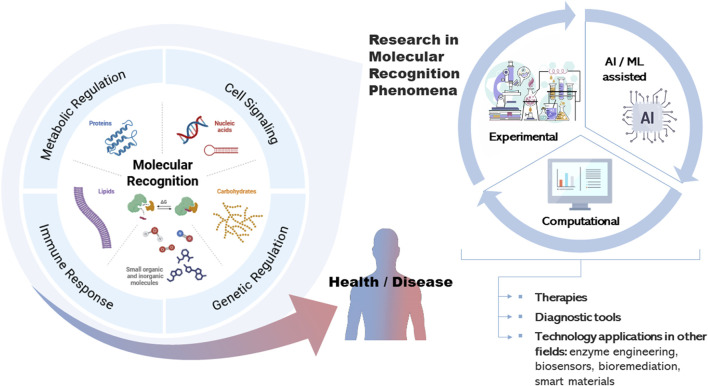
Overview of molecular recognition in biology and medicine. Illustration created with the inputs of Biorender and FreePick.

The significance of molecular recognition has been acknowledged for decades. For instance, Boehr et al. (2006) demonstrated that molecular flexibility and dynamic interactions are key to enzyme activity and regulation. Additionally, Nussinov and Tsai (2013) highlighted the central role of allosteric regulation in modulating signaling pathways under both normal and pathological conditions, thereby opening new avenues for drug discovery. Schreiber et al. (2009) and Baron and McCammon (2013) reviewed the fundamental principles of noncovalent binding, helping to explain how biological systems achieve such high specificity and selectivity. More recently, impaired molecular recognition has been linked to diseases such as cancer and autoimmune disorders, reinforcing its central role in both fundamental biology and clinical medicine (Deng et al., 2025; Jiang et al., 2024).

Recent advances in computational methods are revolutionizing the study of molecular recognition. This Special Topic of Frontiers in Molecular Biosciences focuses on innovative and comprehensive approaches that bridge experimental techniques and computational tools to better understand biomolecular recognition. Artificial intelligence and machine learning are now pivotal in deciphering complex biomolecular interactions, enabling rapid virtual screening, predictive modelling, and the rational design of novel therapeutic agents (D'Hondt et al., 2025; Chen et al., 2018). By integrating experimental and computational methods, we are gaining deeper insights into recognition mechanisms and opening new possibilities for precision medicine and targeted therapies (Figure 1).

With this Research Topic, our initial aim was to explore molecular recognition using combined computational and experimental approaches, especially in contexts relevant to human health. The final collection of four articles, while broader in scope than originally anticipated, offers valuable insights into how molecular mechanisms drive biological complexity and disease. Whilst only one article focuses specifically on structural aspects of molecular recognition, all four contribute to a deeper understanding of biological specificity, molecular function, and their potential in biomedical discovery.

Longshore-Neate et al. examined the conformational flexibility of the nSrc loop in the Src SH3 domain and its modulation by a conserved WX motif found in SH3 domains, contributing to our understanding of protein interactions and specificity in cellular signaling processes. By leveraging experimental approaches combined with structural modelling and molecular dynamics simulations, the study sheds light on how sequence-level conservation can dictate protein-protein recognition dynamics, offering a precise example of molecular recognition in action. Of note, structural modelling was conducted using deep learning techniques via AlphaFold-Multimer, which can predict ligand-bound SH3 domains with remarkable confidence, eliminating the need for traditional docking methods. Indeed, AlphaFold 3 outperforms traditional docking methods across a diverse range of ligands, including proteins, peptides, nucleic acids, and small molecules (Abramson et al., 2024).

Jia et al. presented a comprehensive analysis of Alzheimer’s disease (AD) molecular subtypes based on cuproptosis-related genes. The study employs bioinformatics and machine learning to identify a diagnostic signature, with validation across external datasets and AD mouse models. Amongst the employed tools, several R packages were contemplated for statistical computing of variance, correlation, and weighted networks, such as DESeq2, GSVA, WGCNA, and clusterProfiler. Though not framed as molecular recognition *per se*, it uncovers molecular interactions relevant to neuroinflammation and immune infiltration, which are increasingly understood as recognition-driven processes. A diagnostic model was constructed and validated, providing potential targets for AD treatment. The findings offer novel insights into the pathogenesis of AD.

Soleimani et al. analyzed the immune landscape of chronic liver disease (CLD) using transcriptomics and flow cytometry across three etiologies: chronic viral hepatitis, alcohol-related liver disease, and hepatocellular carcinoma. This work contributes detailed immunophenotyping data and highlights context-dependent shifts in immune cell signatures, offering indirect but significant insight into immune recognition and evasion mechanisms in liver disease. In this study, cell-type deconvolution was achieved using the Dampened Weighted Least Squares (DWLS) algorithm in the R programming language. The Kallisto program was used to quantify and align bulk RNA-Seq.

Jin et al. explored the identification of biomarkers and immune microenvironment features in heart failure through bioinformatics and two machine learning algorithms: Random Forest (RF) and Least Absolute Shrinkage and Selection Operator (LASSO). By integrating gene expression data with immune infiltration profiles, the authors highlight novel markers and cellular contexts relevant to disease progression, pointing toward pathways where molecular recognition events may shape immune–cardiac interactions. The findings suggest potential therapeutic targets for future research in heart failure.

The articles featured in this Special Topic explore the fundamental aspects of molecular recognition but highlight the transformative impact of *in silico* methodologies on advancing the state of the art. Their insights illustrate how the integration of cutting-edge computational tools with classical biochemical techniques is enhancing our mechanistic understanding of cellular processes while revolutionizing strategies for disease intervention. Whether by discovering disease-specific molecular signatures, deciphering the dynamic nature of noncovalent interactions, or employing artificial intelligence for predictive modelling, the articles collectively highlight the power of interdisciplinary innovation to deepen our understanding of biological systems—and ultimately, to drive new diagnostic and therapeutic advances. As the boundaries between technology and biology continue to blur, embracing these interdisciplinary advances is essential for driving future breakthroughs in health sciences.

We extend our sincere gratitude to the contributing authors and peer reviewers for their commitment and insights. We hope this Special Topic will stimulate future research that continues to bridge molecular recognition with emerging technologies and disease-relevant applications.

## References

[B1] Abramson J. Adler J. Dunger J. Evans R. Green T. Pritzel A. (2024). Accurate structure prediction of biomolecular interactions with AlphaFold 3. Nature 630, 493–500. 10.1038/s41586-024-07487-w 38718835 PMC11168924

[B2] Baron R. McCammon J. A. (2013). Molecular recognition and ligand association. Annu. Rev. Phys. Chem. 64, 151–175. 10.1146/annurev-physchem-040412-110047 23473376

[B3] Boehr D. D. McElheny D. Dyson H. J. Wright P. E. (2006). The dynamic energy landscape of dihydrofolate reductase catalysis. Science 313 (5793), 1638–1642. 10.1126/science.1130258 16973882

[B4] Chen H. Engkvist O. Wang Y. Olivecrona M. Blaschke T. (2018). The rise of deep learning in drug discovery. Drug Discov. Today. 23, 1241–1250. 10.1016/j.drudis.2018.01.039 29366762

[B5] Deng X. Sun L. Zhang M. Basavaraj R. Wang J. Weng Y. L. (2025). Biochemical profiling and structural basis of ADAR1-mediated RNA editing. Mol. Cell. 85, 1381–1394.e6. 10.1016/j.molcel.2025.02.017 40101712 PMC11972152

[B6] D'Hondt S. Oramas J. De Winter H. (2025). A beginner's approach to deep learning applied to VS and MD techniques. J. Cheminform. 17, 47. 10.1186/s13321-025-00985-7 40200329 PMC11980327

[B7] Jiang S. Lin X. Wu L. Wang L. Wu Y. Xu Z. (2024). Unveiling the structural mechanisms of nonpeptide ligand recognition and activation in human chemokine receptor CCR8. Sci. Adv. 10, eadj7500. 10.1126/sciadv.adj7500 38306437 PMC10836724

[B8] Nussinov R. Tsai C. J. (2013). Allostery in disease and in drug discovery. Cell. 153, 293–305. 10.1016/j.cell.2013.03.034 23582321

[B9] Schreiber G. Haran G. Zhou H. X. (2009). Fundamental aspects of Protein−Protein association kinetics. Chem. Rev. 109, 839–860. 10.1021/cr800373w 19196002 PMC2880639

